# Proximal Femoral Bone Regeneration After an Uncemented Hydroxyapatite-coated Long-stem in Revision Hip Surgery

**DOI:** 10.2174/1874325001812010125

**Published:** 2018-03-30

**Authors:** José Cordero-Ampuero, Eduardo Garcia-Rey, Eduardo Garcia-Cimbrelo

**Affiliations:** 1 Department of Orthopaedic Surgery and Traumatology, University Hospital La Princesa, Madrid, Spain; 2 Medical School, Universidad Autónoma de Madrid, Madrid, Spain; 3 Department of Orthopaedic Surgery and Traumatology, University Hospital La Paz-IDIPaz, Madrid, Spain

**Keywords:** Hydroxiapatite coated long-stem, Bone regeneration, Osteolytic lesions, Hydroxyapatite, Femoral

## Abstract

**Background:**

Bone remodelling with lateral femoral cortex thinning is a major concern after extensively porous-coated long-stem in revision surgery. Extensive hydroxyapatite coated long-stems were introduced to improve osseointegration, but bone remodelling changes have not been quantified.

**Objective:**

The question of whether bone remodelling changes from extensive hydroxyapatite-coated long stems influence the durability of femoral revision, clinical outcome is assessed in follow-up radiographs.

**Methods:**

Uncemented straight monoblock hydroxyapatite-coated long-stems used in revision hip surgery for aseptic loosening were assessed in a consecutive series of 64 hips (60 patients). Mean follow-up was 8.6 years and the mean age at surgery was 70 years (27-91). The pre-operative bone defect was classified according to Paprosky. Cortical struts were not used in this series. Cortical index and femoral cortical width were measured at three different levels at different periods.

**Results:**

Four patients with pain under level 4 due to stem loosening needed an exchange surgery of their femoral component, but two patients rejected re-surgery. The cumulative probability of not having aseptic loosening was 91.2% (95% confidence interval 73.5-96.9) at 10 years according to Kaplan and Meier. Twenty-seven of 35 osteolytic lesions had disappeared or decreased at the last follow-up. The thickness of the lateral and medial cortex increased over the course of the study at different levels. Increases of femoral cortex thickness were greater in men and in cases with mild bone defects.

**Conclusion:**

Although clinical outcome of the hydroxyapatite-coated long stem in revision surgery is good but not outstanding, most osteolytic lesions heal and the femoral cortex thickness increases at different levels.

## 1 INTRODUCTION

Femoral loosening frequently causes major bone defects, making revision surgery difficult. In revision surgery cementless long-stem prostheses are widely used to create stable stem fixation distal to any deficient bone stock of the proximal femur during revision [1-3]. Although an extensively porous-coated stem has been reported to give good clinical results in revision surgery after more than ten years, severe stress shielding resulting in proximal femur bone loss has been a side-effect that may render further revisions more complex in the long-term outcome of these extensively porous-coated stems in revision surgery. Increased medial femoral cortical thickness and decreased lateral cortical thickness have been reported [4-13]. Series using a hydroxyapatite (HAP) coating with different designs in primary arthroplasty report absence of thigh pain and radiographic ongrowth in most cases [14-16]. In a series of 24 HAP coated Profile stems (DePuy, Warsaw, IN), Hamadouche *et al*. report a mean stem migration of 1.26 mm using EBRA femoral component analysis [17]. Their study showed that with the same stem design, HAP coating enhanced the stability of the femoral stem when compared with grit-blasted stems. Extensive HAP coating of the long stems was introduced to improve osseointegration in revision surgery anad survivorship greater than 95% has been reported after 8-12 years [18, 19].

Bone remodelling changes have been quantitatively described with an extensively porous-coated long-stem [13] but not for extensive HAP coating long-stems. We address the question of clinical and radiographic outcomes, especially focused on bone remodeling changes with a fully HAP coated long-stem in femoral revision, which at least theoretically, may provide proximal as well as distal ongrowth. Femoral radiographs have been retrospectively analyzed in a consecutive series measuring the changes over time in the thickness of the femoral cortex at three levels. The second question we asked was whether patient factors, such as gender, age or intraoperative bone defect, influenced changes in cortical thickness, proximal osteopenia and evolution of osteolytic lesions.

## 2 MATERIALS AND METHODS

**Fig. (1):**
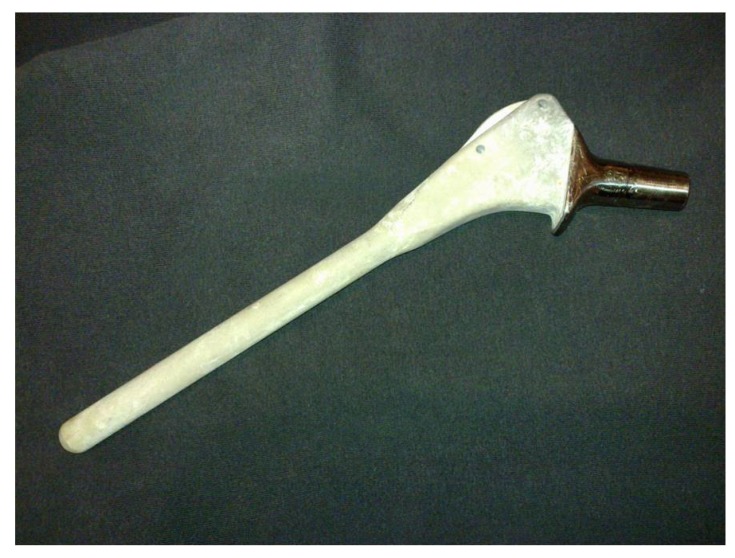
Photograph showing the Furlong extensivly hydroyapatite-coated long-stem used in this series.

Oral and written informed consent was obtained preoperatively from all patients for the hip revision surgery, including the possibility of receiving a fully HAP-coated long stem. This study was retrospective and designed years after these surgeries, so clearly not all patients explicity accepted participation in this specific study. This is a university-hospital, and according to our national laws, all patients have accepted in the informed consent that their clinical records may be used in retrospective research studies. After obtaining Institutional Review Board approval (IRB: PI-885), a retrospective cohort study was done employing data from our prospective institutional database. We followed 62 patients with 66 consecutive revision Furlong-HAP-coated long stems (JRI Instrumentation Ltd. London, UK) implanted in our institution between 1996 and 2012. This stem includes full HAP-coating and follows the diaphyseal fit-and-fill principles to ensure long-term osseointegration. This collared monoblock hip stem is made of titanium alloy (titanium-6-aluminium-4-vanadium) with a smooth surface fully coated with a layer of HAP 400 micrometers in thickness. It has two parts: a cylindrical diaphyseal part that has a rounded point and the metaphyseal part, which is an inverted quadrangular pyramid trunk with almost parallel lateral faces (Fig. **1**). Over that period (1996 to 2014), 173 revision surgeries of a femoral stem were performed in the same Department; the other revision stems used were uncemented Protek Robert Mathys Isoelastic (11 cases), uncemented Surgical Flexfit (17 cases), uncemented modular Exactech Accumatch (4 cases), and cemented Link Lubinus long stem (92 cases).”

A minimum four-year clinical and radio graphic follow-up was required for enrollment in this study. Thus, two hips were lost to follow-up (two patients) before four years. The remaining 64 (60 patients) formed the basis of the follow-up study. There were 35 female and 25 male patients with a mean age of 70 years (range, from 27 to 91). The original diagnosis was primary osteoarthrosis in 50 hips, avascular necrosis of the femoral head in seven, post-traumatic arthritis in three, developmental dysplasia of the hip in two, and rheumatoid arthritis in two. The implants revised were 28 Isoelastic Robert Mathys stems (Protek Bern, Switzerlans), 16 Furlong stems (JRI Instrumentation Ltd. London, UK), 12 cemented stems and 8 another cementless stems. Femoral bone defects were intraoperatively classified according the Paprosky *et al*. criteria [8]: Grade 1 (15 hips), Grade 2 (23 hips), Grade 3A (seven hips), and Grade 3B (19 hips). The revision of the stem was the first revision in 54 hips, the second revision in six hips, and the third in four. The average time between the initial total hip arthroplasty and femoral revision surgery was 10.2 years (range, from 0 to 21). The acetabular component was also revised in 51 hips.The mean follow-up until revision or their latest evaluation for the hips included in this follow-up study has been 8.6 years (range, four to 20 years).

All hips were templated before the surgery to determine the appropriate stem width. In planning the operation one selects an appropriate stem size with a fixation depth of at least 5-7 cm in intact distal dyaphiseal bone [6, 11, 12]. All surgeries were performed by a trained orthopaedic surgeon using a posterolateral approach; an extended transtrochanteric osteotomy was added in nine femurs for stem and cement extraction. The surgeon confirmed the bone defect before reaming the femur. In order to prevent fractures, a prophylactic wire was placed around the proximal femur before impaction [20]. No strut grafts were used in this series. Implant data are detailed in Table **1**. After surgey and depending on their bone defect and press-fit quality, patients spent three to five days with the leg in abduction in bed before being allowed to walk with partial weight bearing for three to six weeks. Antibiotic prophylaxis (1 g cefazolin every six hours) was discontinued at 48 hours post-surgery. Subcutaneous heparin was employed as a routine thrombo-embolic preventive measure under the strict protocol of the hospital’s hematology department until patients were fully mobile.

Patients were clinically evaluated in the outpatient clinic using the Merlè-D'Aubigne-Postel clinical score preoperatively and at 1, 3, 6, 9 and 12 months after revision surgery and then annually until the latest visit or death of the patient. The clinical evaluation assessed pain, walking ability, and joint motion, (range, from 1 to 6) [21]. Patients’ follow-up examination included questioning about pain and its location [22]. Standard anteroposterior and lateral radiographs of the pelvis and the operated femur were made for all patients immediately after the operation, at three, at six and at 12 months, and then annually thereafter. The assessment of cortical index and cortical width based on the postoperative radiographs is a very inaccurate and inconsistent method to evaluate stress shielding changes. In addition, there are other problems inherent to radiological techniques such as positioning of the femur due to the measurement variability produced by femoral rotation when evaluating cortical dimensions [23-25]. To reduce the contributions of each of the potential errors, all postoperative and follow-up radiographs followed the same protocol. The patient was positioned supine, with his/her feet together. The x-ray tube was positioned over the symphysis pubis one meter from and perpendicular to the table. To reduce interobserver error, a single experienced observer made all measurements (JCA). Since 2007 all radiographic controls have been digitalised (General Electrics Centricity): the digital images are marked on a computer screen, and measurement precision is under 0.1 mm. The width of the femoral cortex was measured with the digital tool for measuring linear distances on the General Electrics Centricity apparatus. The known diameter of the femoral head was used as internal reference to correct any variations in magnification. The femur was divided into to the Gruen *et al*. zones [26] and femoral canal filling was measured as the ratio of stem width to intramedullary canal width, at three levels [13]. Radiographic ongrowth was not quantitatively scored but qualitatively assessed on all AP and lateral radiographs: it was defined as the absence of complete radiolucent lines on all Gruen zones, that is, bone tissue could be seen directly apposed on the surface of the femoral stem. The first radiographs after the operation were compared with those made during the follow-up evaluations in order to assess bone remodeling. Three levels at the proximal femur were established on anteroposterior (AP) radiographs as in previous literature [13, 29]: level A was just distal to the inferior margin of the lesser trochanter, level B was 6 cm distal to the inferior margin of the lesser trochanter, and level C was 11 cm distal to the inferior margin of the lesser trochanter. Femoral bone quality and restoration of the femur were quantitatively assessed on follow-up anteroposterior radiographs by calculating the femoral Cortical Index [27, 28] as well as the width of the femoral cortex at levels A, B and C [13, 29]. Stress shielding is not classified here according to the frequently used Engh *et al* criteria [30] because they determines stress shielding area rather than bone loss intensity [9]. The existence of residual osteolytic cavities in the femoral cortex were registered, followed and assessed according to Böhm and Bischel [1]: residual osteolysis was quantitatively evaluated during follow-up (measuring largest and smallest diameters) as increasing defects, constant defects, or osseous restoration. Migration was assessed by measuring the vertical subsidence of the femoral stem according to Callaghan *et al*. [28]. The distance from the tip of the greater trochanter to the most proximal and lateral point of the metaphyseal portion of the stem was very easy to measure in this series because of the geometrical form of this design. Stem subsidence of less than 10 mm was not considered significant. Femoral component fixation was graded following Enghs’ criteria for porous prostheses [31].

### 2.1 Statistical Analysis

Qualitative data are expressed as numbers and percentages with quantitative data being expressed as means with ranges. Qualitative data were compared using the chi-square test or Fisher’s exact test, and quantitative data were compared using the Mann-Whitney U test. Kaplan-Meier curves with 95% confidence intervals (CI) were used for survivorship analysis [32]. Correlations of cortical bone thickness at different levels and independent variables (age, gender, Paprosky classification, and stem diameter) were analysed with Pearson, Spearman, Mann-Whitney, Wilcoxon and Analysis Of Variance (ANOVA, with Bonferroni correction) for repetitive measures tests. These correlations were calculated with the SAS 9.3 software package (SAS Institute, Cary, NC. USA). A p-value of <0.05 was considered statistically significant for all analyses.

## 3 RESULTS

Compared with preoperative and immediate postoperative images, the radiographs at the end of follow-up showed frequent changes in the cortical index and femoral cortex (Table **2**). Average medial and lateral cortical bone thickness, as well as femoral diameter, increased in the metaphyseal area (level A) and in the proximal and more distal diaphyseal femur (levels B and C). After parametric analysis, the only significant correlations appeared between a lower Paprosky type (independent variable) and an increase of lateral cortex thickness at level B (dependent variable) and increase of femoral diameter at levels A and B (dependent variables). According to the analysis of variance (ANOVA, with Bonferroni correction) for repetitive measures tests, the following correlations were significant observed: between male gender and an increased medial cortex thickness at levels B and C, and between a lower Paprosky type and increased femoral diameter at level B; no other associations were significant (Table **2**).

Operative complications included seven intra-operative fractures (11.0%) (2 trochanteric and 5 diaphyseal) treated with cable cerclage. Four patients (6.3%) suffered acute infections, one treated with debridement and three with suppressive antibiotics. Three cups were revised for recurrent dislocations. All these complicated cases were included in the follow-up study.

**Fig. (2):**
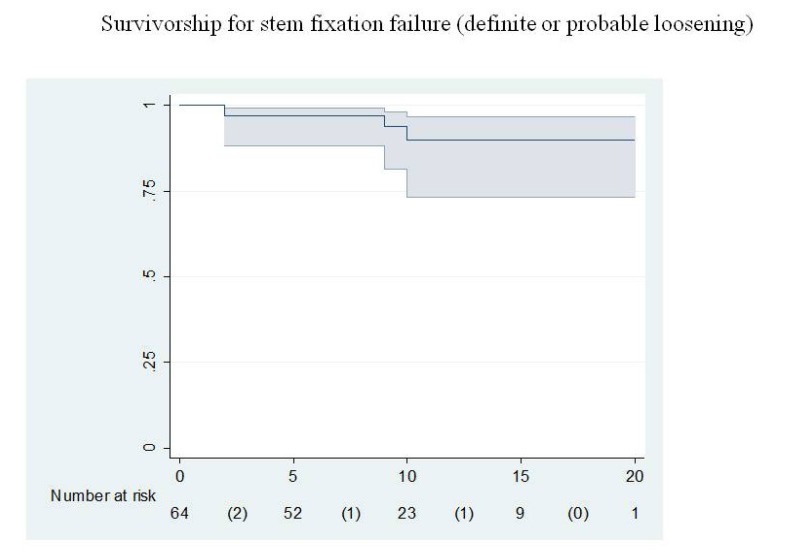
Graph showing the probability of not having aseptic loosening for the whole series. Ranges represent the 95% confidence interval (CI).

The mean preoperative Merle D’Aubigné and Postel scores were 2.0 for pain, 2.1 for function, and 2.2 for range of motion.The mean scores at the latest follow-up study were 4.2 for pain, 4.0 for function, and 4.3 for range of motion. Level 3 pain was found in loosened and rerevised stems. All patients reporting pain referred to distal thigh pain. Frequently, patients had difficulties in flexing their knees during the first weeks after surgery, but they had recovered knee flexion at the three month review. Four patients with pain under level 4 due to stem loosening needed an exchange surgery for their femoral component, but two patients rejected re-surgery. So, only two stems were revised for aseptic loosening. The cumulative probability of not having aseptic loosening was 91.2% (95% confidence interval 73.5-96.9) at 10 years according to Kaplan and Meier (Fig. **2**).

We did not observe radiolucent lines in any Gruen zone in 53 stems. Radiolucent lines were incomplete and limited to Gruen zones 1 and 7 in seven stems. The only cases with complete lucent lines in all Gruen zones were the four stems diagnosed with loosening (two of them re-revised and the other two rejected additional surgery).

All transtrochanteric osteotomies healed uneventfully. The anteroposterior radiograph showed the stem tip to be centered in all hips, those operated by a posterolateral as well as those operated by a transtrochanteric approach. However, eight (14.0%) of the 57 patients operated *via *a posterolateral approach presented an impingement of the tip of the stem on the anterior cortex, resulting in an average loss of 18.0% of the femoral cortex (range, 10 to 22.0%). When an extended transtrochanteric osteotomy was done the stem never impinged on the anterior cortex. No fractures were detected at the tip of the stem.

The mean radiographic non-progressive stem subsidence was 3.5 mm and 6.0% of stems subsided more than 10 mm. These radiographically unstable stems presented a preoperative bone defect classified as Paprosky type II in 2 hip, type IIIA in 1, and type IIIB in 1.

Residual osteolysis was diagnosed after revision surgery in 35 hips. The approximate surface of the osteolytic lesion in each radiographic control is calculated multiplying the largest by the smallest diameter [1]: the comparison of these figures is used to judge qualitatively if the lesion is increasing, constant or restoring. According to this evaluation, at the end of follow-up these osteolytic lesions has disappeared in 15 of these 35 hips (42.9%), decreased in size in 12 (34.3%), remained similar in five (14.3%), and increased in size in three (8.6%) cases. New osteolysis developed in seven of 64 assessed hips (10.9%). Residual osteolysis was not present after revision surgery and remained absent in 20 cases at the end of follow-up.

## 4 DISCUSSION

Bone regeneration is difficult to evaluate radiographically but definite changes do seem to have occured in the proximal femoral cortex, while femoral diameter increased in the metaphyseal area (level A) and in the proximal and more distal diaphyseal femur (levels B and C), which is significantly related to gender and/or Paprosky preoperative defect (Table **2**). The full HAP coating may contribute to this bone regeneration. This evolution is not the same a reported for other revision stems in which bone increased at the medium level but stayed similar at the distal level using a tapered-fluted grit-blasted revision stem [2, 35]; and the cortical index and lateral cortex decreased with an extensively porous-coated stem [13]. Distal fixation promotes proximal stress-shielding [9] but no significant stress-shielding has also been reported [36, 34, 38]. No similar quantitative measurements have been published previously using the Furlong-HAP revision stems: Raman *et al*. and Trikha *et al*. [18, 19] have only reported “endosteal bone formation”, in their subjective qualitative evaluation. Grafts were not used in our series and although a lateral cortical strut graft may well have improved results in hips with thin lateral cortices and massive femoral bone loss [37], we agree with Nadaud *et al*. that allografts make it difficult to achieve a tight press fit in the host bone [12].

Our clinical results using a revision Furlong-HAP-coated long stem are good in most hips in this series. Thigh pain was less common in patients with stems that achieved fixation with radiographic bone ongrowth [8]. The re-revision rate of the stem in this series was better than some studies (8.6%-11.0%) [33], similar to others (6.5% loosening, 4.3% revision [2, 36], but worse than other series (1.3-3.9%) [18, 19, 34, 35]. These discrepancies can be explained by the complication rate. Poor results and an especially high number of complications could be attributed to a lack of surgical experience instead of blaming the specific and nowadays obsolete characteristics of the stem, in comparison with newer designs. There were seven intra-operative fractures (11.0%), a higher prevalence than in other series [18, 19]. As rotational stability of this stem is based exclusively on metaphyseal fit-and-fill, the increase in size of straight reamers facilitates trochanter fracture and distal perforation, especially in short femurs. Three hips were revised with a cup exchange for recurrent dislocation. These figures are well over the 6.0-7.7% rate reported [18, 19]. Other revision series with uncemented stems report 0.0% [34] to 4.3% [36]. The wide cervico-diaphyseal angle and low offset of this design contribute to this complication. A second explanation is the lack of acetabular revision in some cases, when old polyethylenes were maintained. Patient age does not seem to have a negative effect on the postoperative clinical results since mean patient age in this series was 70 years old.

Radiographic bone ongrowth was diagnosed in 94.0% of the stems, and unstable fixation in four stems, which compares favourably with some porous-coated designs [4, 9, 10], but is worse than the 1-4.0% reported with other uncemented revision implants [2, 13, 16, 18, 19, 34, 38]. The four unstable patients needed an exchange surgery for their femoral component, but two of them rejected re-surgery. Average stem subsidence (3.5 mm) in this series was worse than that published previously for the same stem [18]. Residual osteolysis was diagnosed after revision surgery in 35 cases and at the end of follow-up these osteolytic lesions disappeared in 15 of those 35 hips (42.9%). The evolution of residual osteolysis was not analysed in one of the previous series [18, 19]. Other published studies with other designs only report no diaphyseal osteolysis and no increasing focal defects at the end of follow-up [10, 35].

Our study has some limitations. One is the small cohort and short follow-up resulting in some incomplete clinical outcome data. However, we believe that this follow-up is sufficient to support our conclusions. Our data were obtained from a prospective clinical and radiographic follow-up data base and were collected retrospectively for this study. We did not randomise these components with other designs for comparisons. The Merle d'Aubigne score does not only evaluate the femoral side, rather it assesses the global function of the hip, and is also related to other parameters including acetabular side, previous procedures, and abductor mechanism. We performed no analysis of inter-intraobserver variability for the radiographic measurements. The investigating surgeon studied the radiographs and entered the data on forms on the day of the clinic visit. These detailed measurements were made by another (unblinded) reviewer at a different time. Thus, some bias may have been introduced in the radiographic assessment. We recognize our radiographic measurements of variations in femoral cortex thickness may not be sufficiently reliable, but clinically and radiographically we do see bone remodelling changes in the femoral cortex.

## CONCLUSION

In conclusion, the Furlong straight monoblock HAP-coated long stem in revision surgery allows us to solve difficult cases with major proximal bone defects that would be difficult to revise with other methods. Clinical outcome is good but not outstanding compared with other designs. Radiographic bone fixation is frequent, but unstable fixation was more frequent when there were major bone defects. Contrary to other porous long-stems, most osteolytic lesions heal and cortical thickness increases at different levels. Alternative methods of reconstruction, such as an impaction grafting procedure, should be considered in young patients who will probably require a new femoral revision at some point in their future. The Furlong extensively HAP-coated long-stem favours the proximal bone regeneration in femoral revision surgery.

## Figures and Tables

**Table 1:** Implant data.

Size of component	Number of cases (%)
Stem length	-
200 mm	42 (65.6%)
250	22 (34.4%)
Stem diameter	-
10 mm	4 (6.2%)
12 mm	17 (26.6%)
14 mm	17 (26.6%)
16 mm	22 (34.4%)
18 mm	18 (28.1%)
Diameter of femoral head	-
28 mm	42 (65.6%)
32 mm	22 (34.4%)

**Table 2:** Variations in mm in the femoral cortex at levels A, B and C and the femoral diameter in the hips included in the follow-up study, mean +standard deviation, and correlations of cortical bone thickness at different levels and independent variables, p value.

-	**Post-operative**	**Last** **follow-up**	**Age +**	**Stem+**	**Paprosky [** **8** **]+**	**Gender** **++**	**Age** **+++**	**Stem** **+++**	**Paprosky [** **8** **] +++**	**Gender+++**
**Level A**	-	-	-	-	-	-	-	-	-	-
Medial cortex	7.5 + 4.1	8.3 + 4.3	0.237	0.298	0.891	0.060	0.412	0.131	0.617	0.172
Lateral cortex	7.3 + 3.9	7.9 + 5.2	0.558	0.382	0.674	0.326	0.455	0.174	0.791	0.090
Femoral diameter	39.6 + 7.0	40.4 + 7.3	0.933	0.576	0.018*	0.201	0.752	0.967	0.052	0.689
**Level B**	-	-	-	-	-	-	-	-	-	-
Medial cortex	7.3 + 3.5	7.4 + 3.5	0.608	0.310	0.556	0.191	0.706	0.838	0.432	0.033*
Lateral cortex	6.9 + 2.5	7.6 + 3.9	0.642	0.062	0.030*	0.738	0.898	0.233	0.235	0.961
Femoral diameter	33.7 + 5.4	34.9 + 6.0	0.433	0.352	0.020*	0.848	0.577	0.945	0.046*	0.607
**Level C**	-	-	-	-	-	-	-	-	-	-
Medial cortex	7.4 + 3.0	7.7 + 3.3	0.948	0.081	0.396	0.063	0.866	0.450	0.398	0.021*
Lateral cortex	6.2 + 2.3	6.3 + 2.4	0.891	0.095	0.054	0.107	0.946	0.105	0.472	0.106
Femoral diameter	31.9 + 5.0	32.8 + 4.6	0.970	0.860	0.846	0.382	0.769	0.475	0.475	0.117
+Pearson; ++, Mann-Whitney and Wilcoxon; +++, ANOVA; * only Statistically significant correlations										

## LIST OF ABBREVIATIONS

ANOVA: = Analyis Of Variance
CI: = Confidence Interval
DEXA: = Dual-Energy X-ray Absortiometry
HAP: = Hydroxyapatite
